# Nitidine Chloride Inhibits SIN1 Expression in Osteosarcoma Cells

**DOI:** 10.1016/j.omto.2019.01.005

**Published:** 2019-02-05

**Authors:** Hui Xu, Tong Cao, Xiaoqing Zhang, Ying Shi, Qing Zhang, Shuo Chai, Li Yu, Guoxi Jin, Jia Ma, Peter Wang, Yuyun Li

**Affiliations:** 1 Department of Laboratory Medicine, School of Laboratory Medicine, Bengbu Medical College, Bengbu, Anhui 233030, China; 2 Department of Clinical Laboratory, The First Affiliated Hospital of Bengbu Medical College, Bengbu, Anhui 233004, China; 3 Research Center of Clinical Laboratory Science, Bengbu Medical College, Bengbu, Anhui 233030, China; 4 Department of Orthopedics, The Center Hospital of Bengbu, Bengbu, Anhui 233030, China; 5 Department of Endocrinology, The First Affiliated Hospital of Bengbu Medical College, Bengbu, Anhui 233030, China; 6 Department of Biochemistry and Molecular Biology, School of Laboratory Medicine, Bengbu Medical College, Bengbu, Anhui 233030, China

**Keywords:** osteosarcoma, nitidine chloride, SIN1, growth, apoptosis, invasion

## Abstract

Nitidine chloride (NC) has been demonstrated to exert a tumor-suppressive function in various types of human cancers. However, the detailed mechanism of NC-mediated anti-tumor effects remains elusive. It has been reported that SIN1, a component of mTORC2 (mammalian target of rapamycin complex C2), plays an oncogenic role in a variety of human cancers. Therefore, the inhibition of SIN1 could be useful for the treatment of human cancers. In this study, we explored whether NC triggered an anti-cancer function via the inhibition of SIN1 in osteosarcoma (OS) cells. An MTT assay was performed to measure the effect of NC on the cell growth of osteosarcoma cells, and flow cytometry was used to detect the apoptotic rate of the cells after NC treatment. The expression of SIN1 was detected by western blotting. Wound-healing assay and Transwell chamber invasion assay were conducted to analyze the motility of osteosarcoma cells following NC exposure. We found that exposure to NC led to the inhibition of cell growth, migration, and invasion and the induction of apoptosis. Mechanistically, we found that NC inhibited the expression of SIN1 in osteosarcoma cells. Overexpression of SIN1 abrogated the inhibition of cell growth and motility induced by NC in osteosarcoma cells. Our results indicate that NC exhibits its tumor-suppressive activity via the inhibition of SIN1 in osteosarcoma cells, suggesting that NC could be a potential inhibitor of SIN1 in osteosarcoma.

## Introduction

Osteosarcoma (OS) is one of the common primary malignant bone tumors, which often occurs in adolescents and young adults.^1^ Currently, the 5-year survival rates have improved to 60%–70% in patients with localized osteosarcoma after multidisciplinary treatments.^2^ However, the 5-year survival rate in osteosarcoma patients with metastatic disease is only about 20%–30%.^3^ Although treatment of osteosarcoma has been improved, metastatic osteosarcoma patients often have poor prognoses and they relapse.^4^ Discovery of new therapeutic agents is pivotal to improving the treatment outcome in osteosarcoma patients.

The mammalian target of rapamycin (mTOR) as a serine or threonine protein kinase has been reported to contribute to the development and progression of human cancers, including osteosarcoma.^5^ It has been known that mTOR belongs to the phosphoinositide-3-kinase (PI3K)-related kinase family, which controls multiple cellular processes such as cell growth, apoptosis, and metabolism.^6^ The mTOR complexes include two distinct parts, mTORC1 and mTORC2. mTORC1 includes five components: mTOR, mammalian lethal with Sec13 protein 8/G protein β subunit-like protein (mLST8/GβL), regulatory-associated protein of mTOR (Raptor), proline-rich Akt substrate of 40 kDa (PRAS40), and DEP domain-containing mTOR-interacting protein (DEPTOR).^6^ mTORC2 consists of six components: mTOR, Rapamycin-insensitive companion of mTOR (Rictor), DEPTOR, mLST8/GβL, protein observed with Rictor-1/proline-rich protein 5 (PROTOR), and mSIN1 (also named as mitogen-activated protein kinase-associated protein 1 [MAPKAP1]).^6^ It has been demonstrated that mTOR is a key sensor for metabolic and nutrient stresses to control cellular metabolism, cellular growth, and survival.^7^ SIN1 phosphorylation enhanced the activity of mTORC2,^8^ suggesting an important role of SIN1 in cancer development and progression. Therefore, the inhibition of SIN1 may be a promising strategy for cancer treatment.

Nitidine chloride (NC), a natural bioactive phytochemical alkaloid, was originally discovered to exhibit anti-fungal, anti-inflammatory, and anti-oxidant functions.^9^ In recent years, NC was reported to exert its anti-tumor activity in various types of human malignant cancers.^10^ One study has demonstrated that NC inhibited cell proliferation and induced apoptosis in MG63 cells.^11^ However, the mechanism of NC-mediated anti-cancer activity in osteosarcoma has not been fully elucidated. Thus, in this study, we aimed to investigate the effects of NC on cell growth, apoptosis, migration, and invasion in osteosarcoma cells. We also determined whether NC-induced tumor suppression in osteosarcoma cells is through the regulation of SIN1.

## Results

### NC Inhibits Osteosarcoma Cell Proliferation

**Figure 1:**
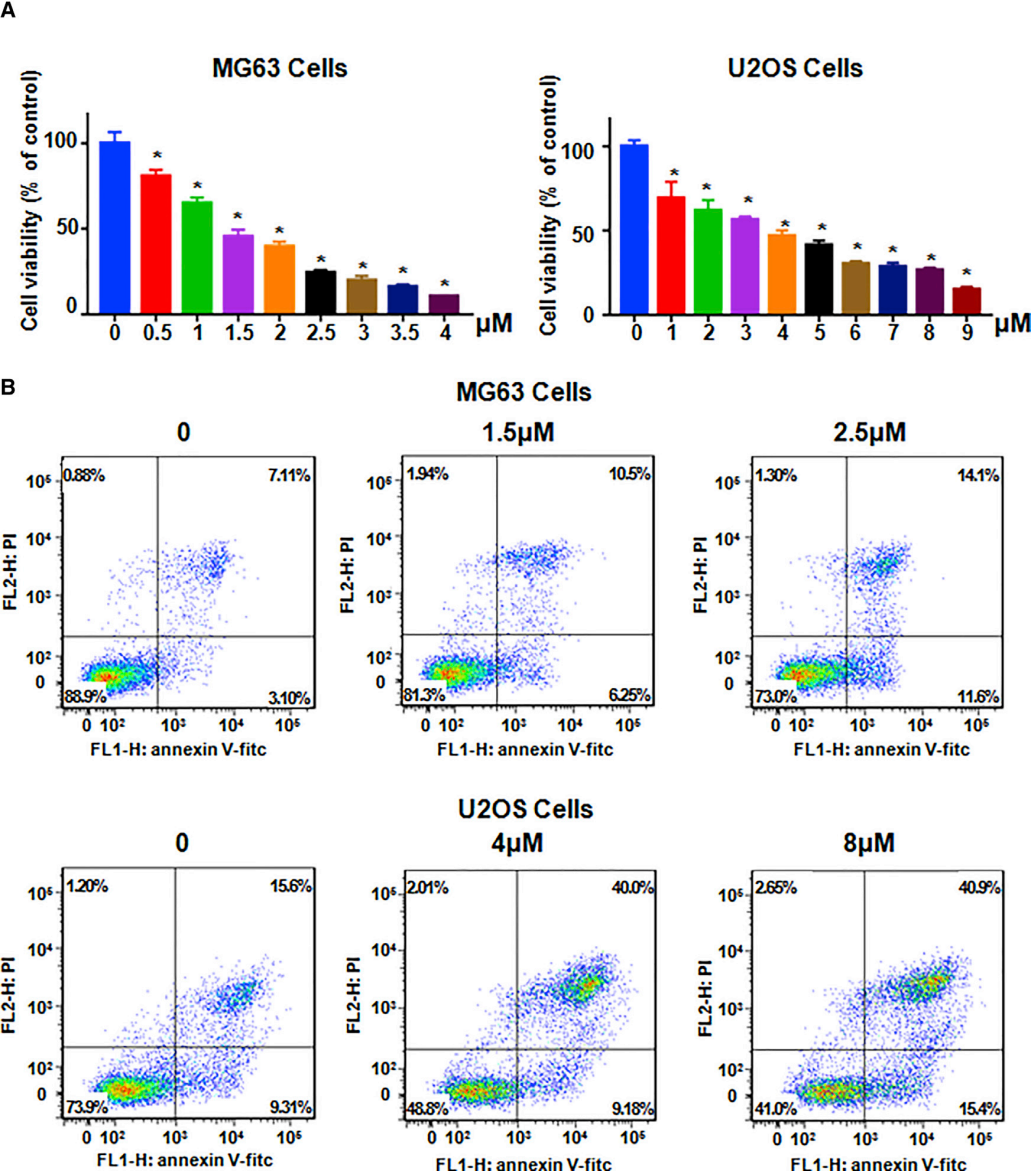
Effect of NC on Osteosarcoma Cell Growth and Apoptosis (A) The effect of NC on cell growth in MG63 cells and U2OS cells was detected by MTT assay after treatment with NC for 72 h. *p < 0.01 compared with control group (DMSO treatment group). (B) Cell apoptosis in MG63 cells and U2OS cells was examined by Annexin V-FITC/PI method after NC treatment for 48 h.

To investigate whether NC treatment could suppress osteosarcoma cell proliferation, we used an MTT (3-4,5-dimethylthiazol-2,5-diphenyltetrazolium bromide) assay to measure the cell growth inhibition in MG63 cells and U2OS cells treated with different concentrations of NC for 72 h. Our MTT results showed that cell growth was significantly inhibited by NC in a dose-dependent manner (Figure 1A). Specifically, we found theat 1.5 and 4 μM NC could suppress about 50% cell growth in MG63 cells and U2OS cells, respectively. Therefore, NC inhibited osteosarcoma cell proliferation.

### NC Induces Cell Apoptosis

To explore whether NC treatment could trigger cell apoptosis, MG63 cells and U2OS cells were treated with different doses of NC for 48 h, and then the collected cells were measured by Annexin V-fluorescein isothiocyanate (FITC) and propidium iodide (PI) assay. Our results showed that NC treatment caused cell apoptosis obviously (Figure 1B). Moreover, the percentage of apoptotic cells was from 10.21% to 25.70% in MG63 cells treated with 2.5 μM NC and from 24.91% to 56.3% in U2OS cells following 8-μM NC exposure. Altogether, NC treatment stimulated cell apoptosis in osteosarcoma cells.

### NC Inhibits Cell Migration and Invasion

**Figure 2:**
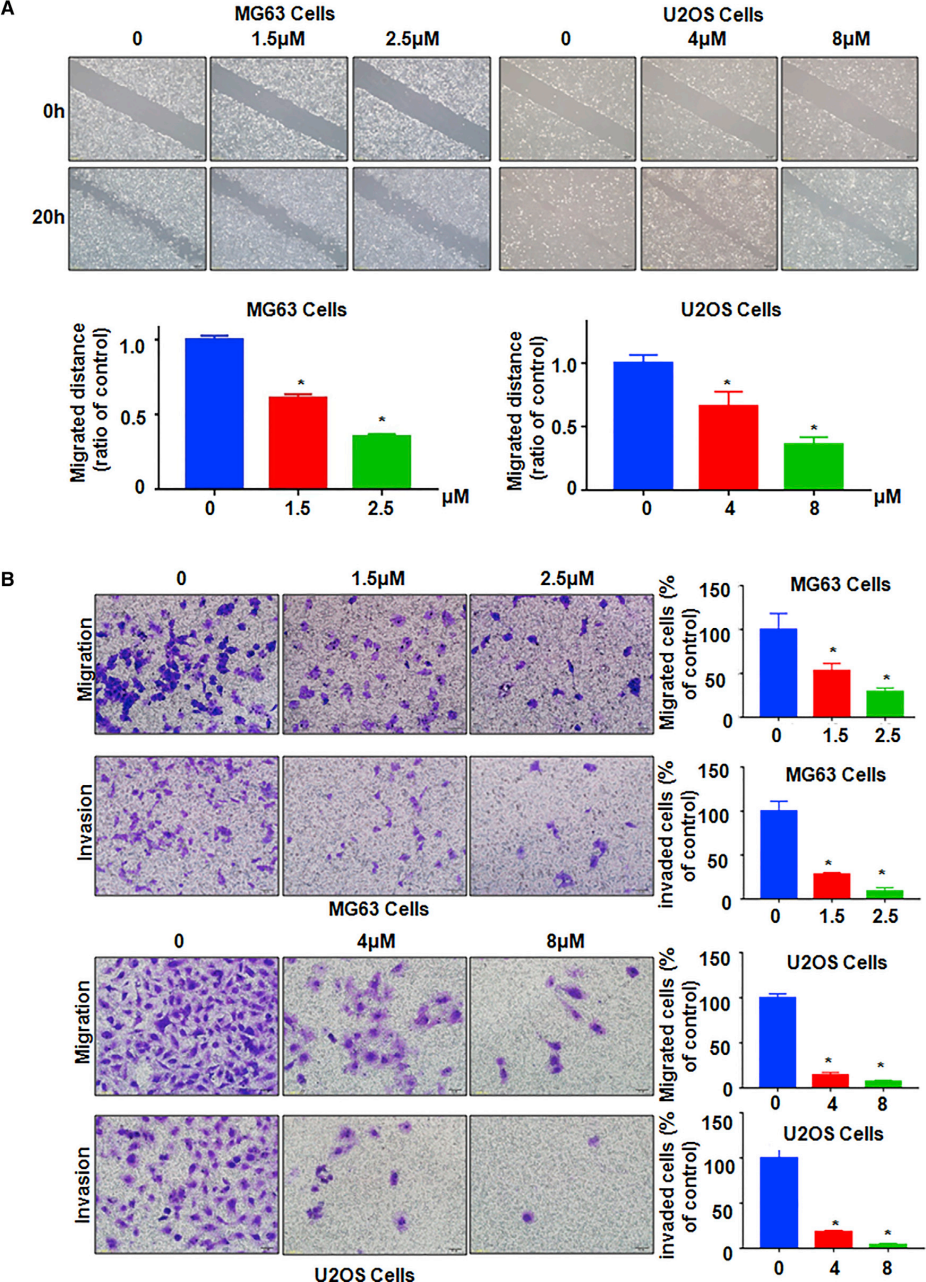
Effect of NC on Cell Migration and Invasion (A) Top: cell motility was detected using a wound-healing assay in MG63 cells and U2OS cells after NC treatment. Bottom: quantitative results are illustrated for the top image. *p < 0.01 versus control group (DMSO treatment group). (B) Left: cell migration and invasion were determined using Transwell assay with or without Matrigel in MG63 cells and U2OS cells after NC treatment for 24 h. Right: quantitative results are illustrated for the left image. *p < 0.01 compared with control.

To dissect whether NC could affect cell migration and invasion, we selected the scratch wound-healing assay and Transwell assay to measure these effects. Our wound-healing assay results showed that NC treatment significantly inhibited cell migration in a dose-dependent manner in both osteosarcoma cell lines (Figure 2A). Our transwell assay results demonstrated that NC treatment profoundly decreased the invasive capacity of MG63 and U2OS cells (Figure 2B). Taken together, NC treatment inhibits cell motility activity.

### NC Decreases SIN1 Expression

**Figure 3:**
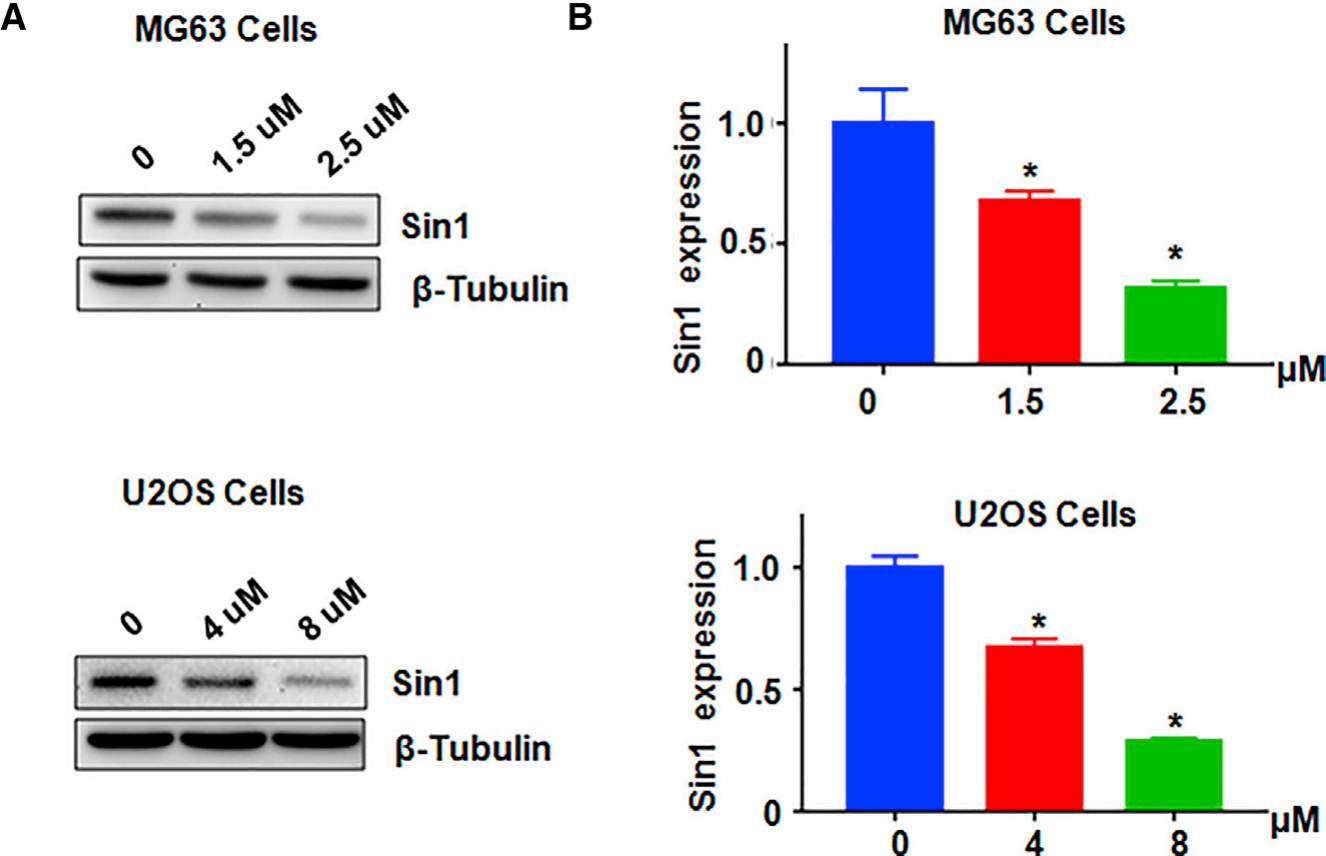
NC Inhibits SIN1 Expression (A) The expression of SIN1 was measured by western blotting in MG63 cells and U2OS cells after NC treatment. (B) Quantitative results are illustrated for (A). *p < 0.01 compared with control (DMSO treatment group).

Sin1 has been reported to be an oncoprotein in tumorigenesis. Thus, we wondered whether NC could decrease SIN1 expression in osteosarcoma cells. We used western blotting to measure SIN1 levels in MG63 cells and U2OS cells after treatments with different concentrations of NC. Our western blotting results showed that SIN1 expression was significantly suppressed by NC in both osteosarcoma cell lines (Figures 3A and 3B). This result indicated that NC downregulated SIN1 expression in osteosarcoma cells.

### Overexpression of SIN1 Facilitates Cell Proliferation and Neutralizes the Effects of NC on Cells

**Figure 4:**
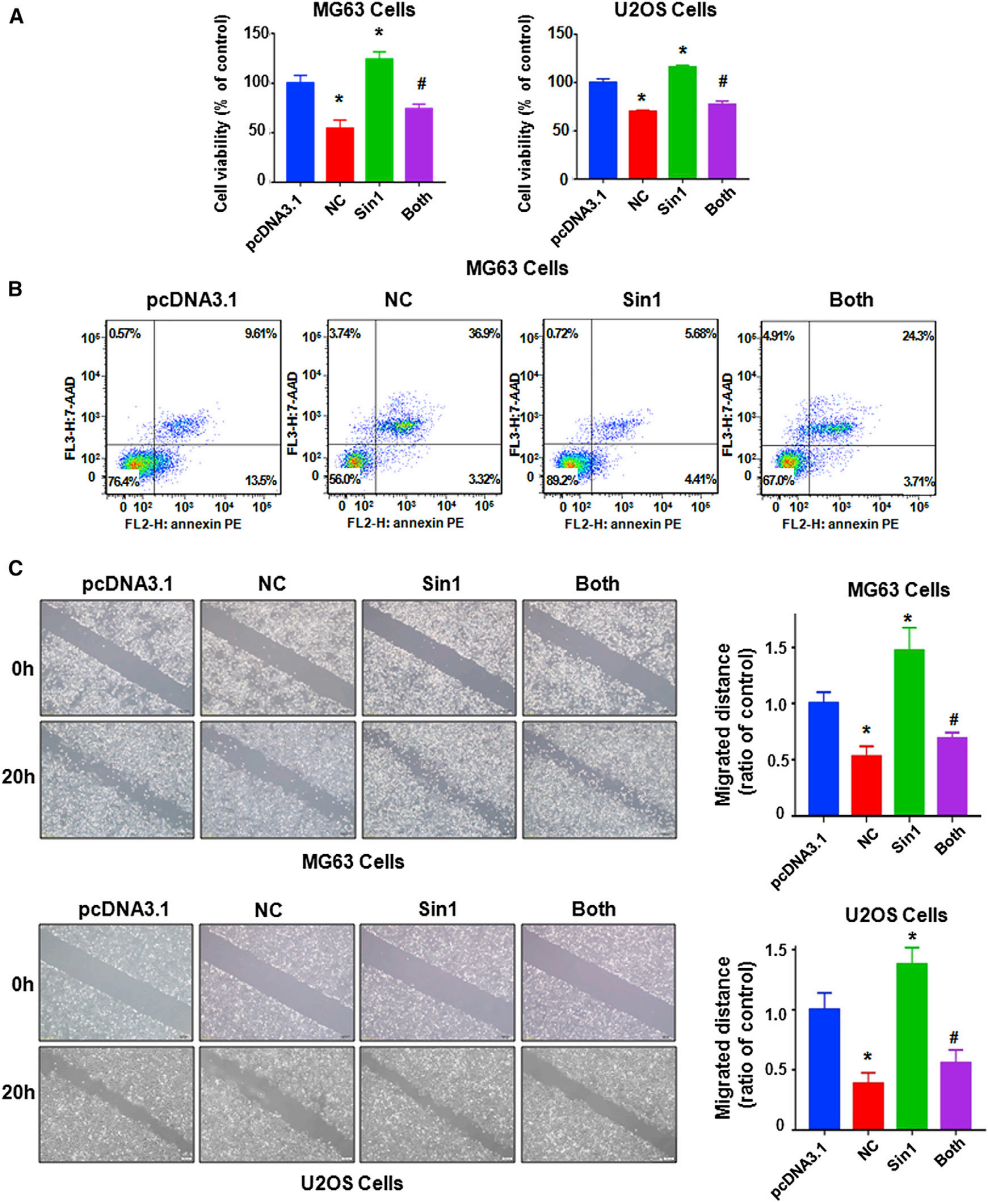
Effects of SIN1 Overexpression on Cell Growth Apoptosis and Migration (A) The effect of SIN1 overexpression in combination with NC treatment on osteosarcoma cell growth by MTT assay. Control, pcDNA3.1; MG63, 1.5 μM NC; U2OS, 4 μM NC; Both, Sin1 cDNA^+^NC. (B) Cell apoptosis in MG63 cells and U2OS cells was detected by flow cytometry after Sin1 cDNA transfection and NC treatment. (C) Left: cell migration was measured by a wound-healing assay after SIN1 cDNA transfection and NC treatment. Right: quantitative results are illustrated for the left image. *p < 0.01 compared with control; ^#^p < 0.05 compared with NC treatment alone or SIN1 cDNA transfection alone.

To determine the effect of SIN1 in osteosarcoma cells, MG63 cells and U2OS cells were transfected with SIN1 cDNA or empty vector as control. Our results showed that the overexpression of SIN1 enhanced cell growth in both osteosarcoma cell lines and partly abrogated cell growth inhibition by NC (Figure 4A). Moreover, the overexpression of SIN1 significantly reduced cell apoptosis and abrogated NC-induced apoptosis in MG63 cells (Figure 4B). Therefore, these data suggest that SIN1 plays an important oncogenic role in NC-induced cell growth inhibition and apoptosis.

**Figure 5:**
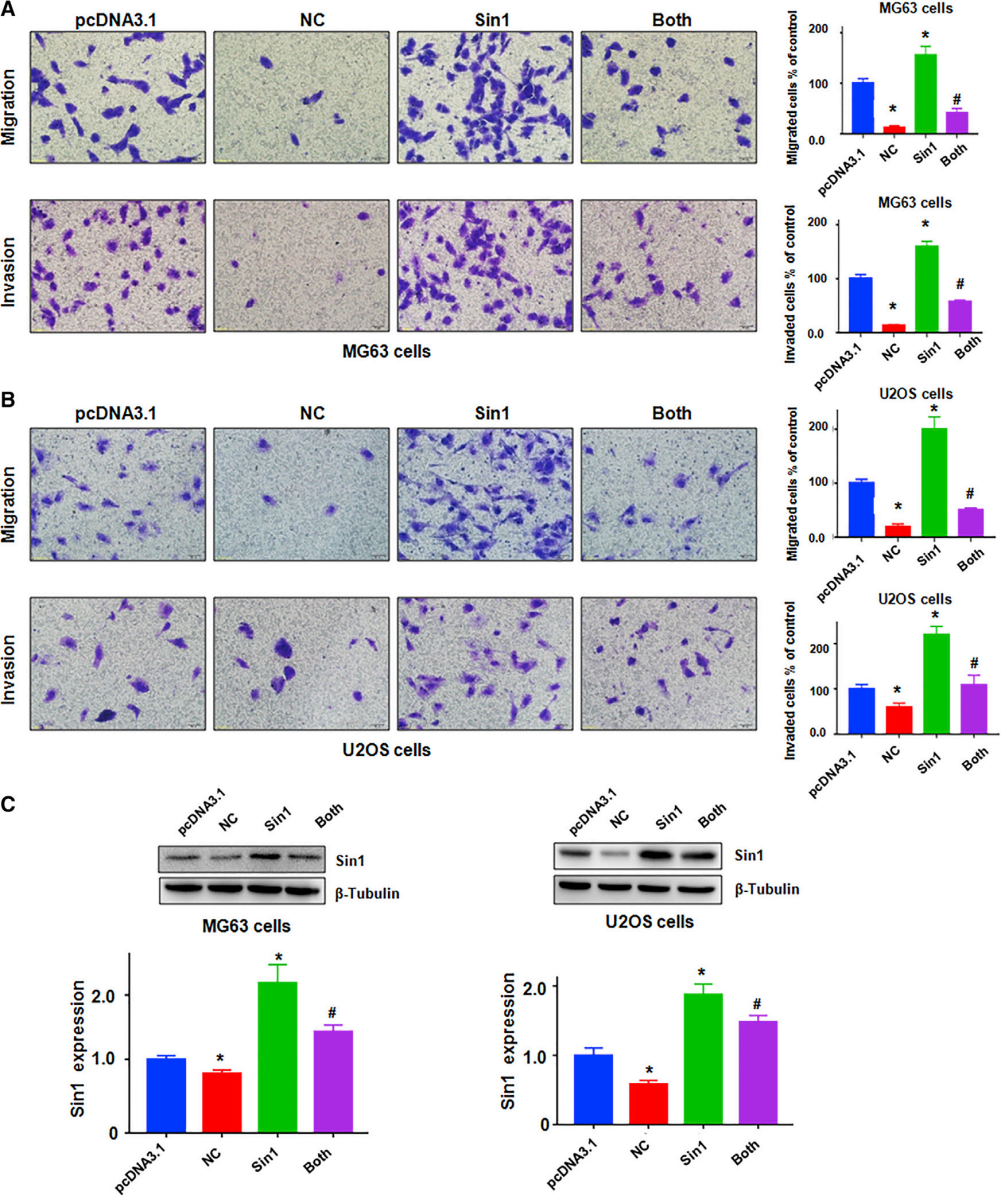
Overexpression of Sin1 Reinforces Osteosarcoma Cell Invasion (A and B) Left: cell migration and invasion were detected by a Transwell chamber assay with or without Matrigel in MG63 cells (A) and U2OS cells (B) after SIN1 cDNA transfection and NC treatment. Right: quantitative results are illustrated for the left image. Control, pcDNA3.1; MG63, 1.5 μM NC; U2OS, 4 μM NC; Both, SIN1 cDNA^+^NC. (C) Top: the expression of SIN1 was detected by western blotting in osteosarcoma cells with SIN1 cDNA transfection and NC treatment. Bottom: quantitative results are illustrated for the top image. *p < 0.01 compared with control; ^#^p < 0.05 compared with NC treatment alone or Sin1 cDNA transfection alone.

Subsequently, we used the wound-healing assay and transwell assay to further explore whether SIN1 is involved in cell motility in osteosarcoma cells. Our results showed that SIN1 overexpression enhanced cell migration and attenuated the inhibitory effects of NC on cell migration abilities in MG63 cells and U2OS cells (Figure 4C). Next, the Transwell assay was applied to verify the function of SIN1 in the invasive potential of osteosarcoma cell lines. As we expected, the overexpression of SIN1 promoted cell invasion in both MG63 cells and U2OS cells (Figures 5A and 5B). Moreover, SIN1 overexpression abrogated the NC-induced cell invasion inhibitory effects (Figures 5A and 5B). We also detected the expression of SIN1 in osteosarcoma cells after NC treatment in combination with SIN1 cDNA transfection. We found that SIN1 overexpression partially reversed the inhibition of NC on SIN1 (Figure 5C). These results suggested that NC exerted its anti-tumor function by the inhibition of SIN1 in osteosarcoma cells.

### Downregulation of SIN1 Enhances the Anti-cancer Activity of NC

**Figure 6:**
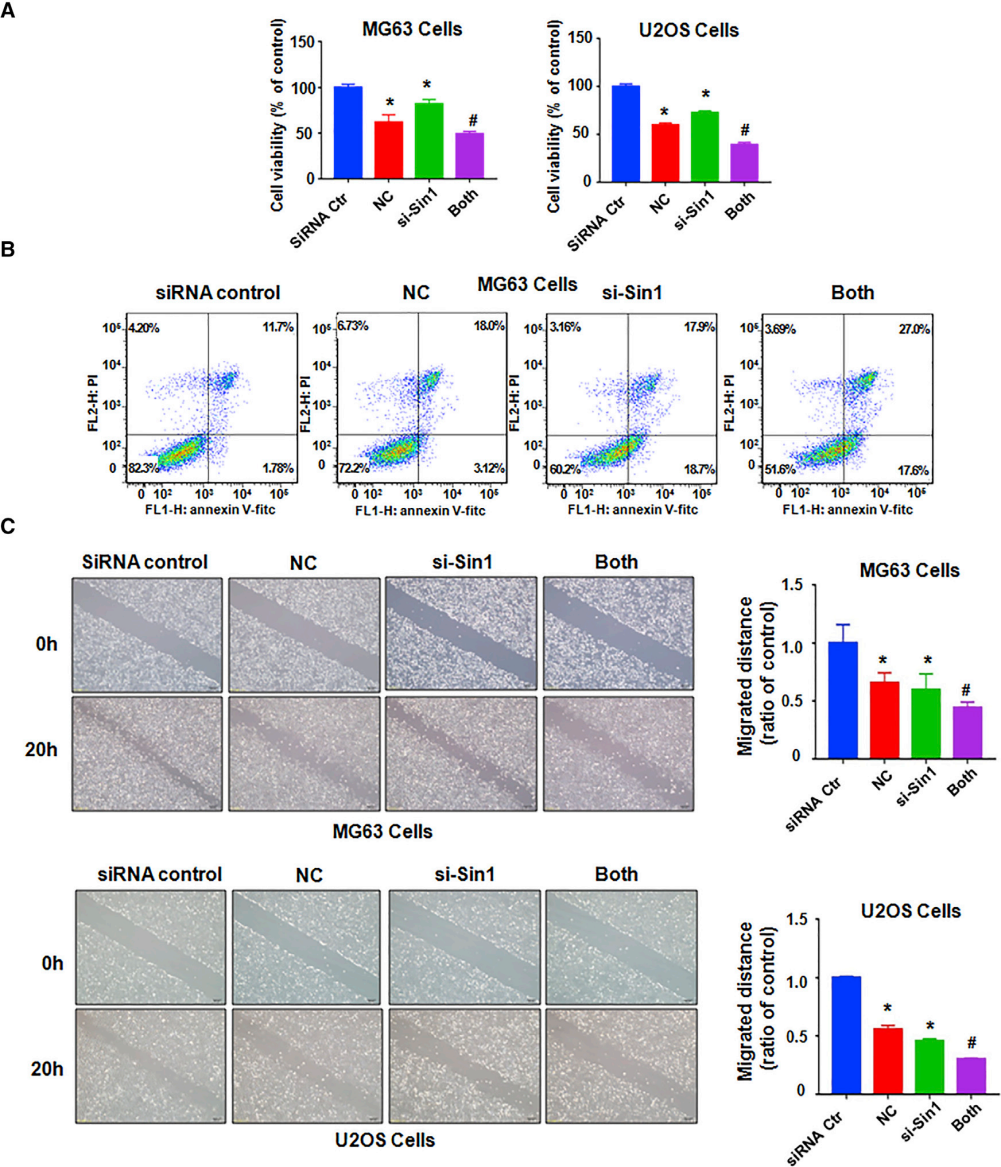
Downregulation of SIN1 Inhibits Cell Growth and Migration and Promotes Cell Apoptosis in Osteosarcoma Cells (A) The effect of SIN1 down-expression in combination with NC treatment on osteosarcoma cell proliferation was detected by MTT assay. siRNA Ctr, siRNA control; MG63, 1.5 μM NC; U2OS, 4 μM NC; siRNA, SIN1 siRNA; Both, SIN1 siRNA^+^NC. (B) Cell apoptosis was tested via flow cytometry in MG63 cells after NC exposure and SIN1 siRNA transfection. (C) Left: cell migration was measured by a wound-healing test after SIN1 siRNA transfection and NC treatment. Right: quantitative results are illustrated for the left image. *p < 0.01 compared with control; ^#^p < 0.05 compared with NC treatment alone or SIN1 siRNA transfection alone.

To further explore whether SIN1 is involved in cell sensibility to the cytotoxicity of NC, both osteosarcoma cells were transfected with SIN1 small interfering RNA (siRNA). The results showed that the downregulation of SIN1 obviously inhibited cell growth (Figure 6A). Moreover, the downregulation of SIN1 in combination with NC induced a higher cell growth inhibition compared with NC alone or siRNA tansfection alone (Figure 6A). Furthermore, we found that NC treatment plus SIN1 siRNA transfection resulted in a higher percentage of apoptotic cells compared with each alone in MG63 cells (Figure 6B).

**Figure 7:**
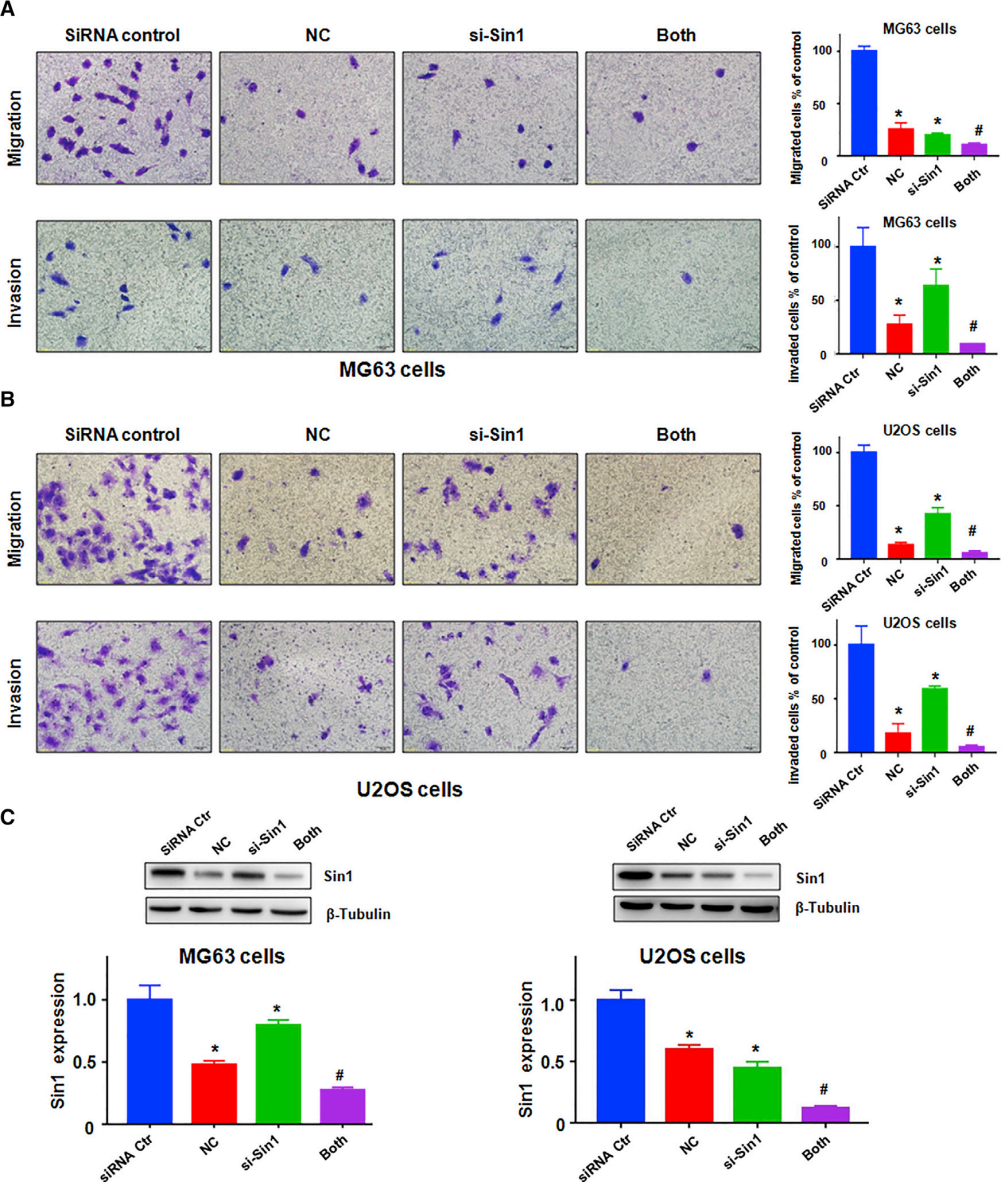
Downregulation of SIN1 Inhibits Osteosarcoma Cell Invasion (A and B) Left: cell migration and invasion were detected by a Transwell chamber assay with or without Matrigel in MG63 cells (A) and U2OS cells (B) after SIN1 siRNA transfection and NC treatment. Right: quantitative results are illustrated for the left image. siRNA Ctr, siRNA control; MG63, 1.5 μM NC; U2OS, 4 μM NC; siRNA, SIN1 siRNA; Both, SIN1 siRNA^+^NC. (C) Top: the expression of SIN1 was detected by western blotting in osteosarcoma cells with SIN1 siRNA transfection and NC treatment. Bottom: quantitative results are illustrated for the top image. *p < 0.01 compared with control; ^#^p < 0.05 compared with NC treatment alone or SIN1 siRNA transfection alone.

Our wound-healing assay showed that down-expression of SIN1 suppressed cell migration in both cell lines (Figure 6C). Our transwell assay demonstrated that downregulation of SIN1 inhibited cell invasion in both cell lines (Figures 7A and 7B). More importantly, NC plus SIN1 siRNA led to a greater degree of inhibition compared with NC alone or siRNA transfection alone (Figures 7A and 7B). Our western blotting analysis results showed that SIN1 siRNA transfection reinforced the inhibition of SIN1 induced by NC in osteosarcoma cells (Figure 7C). These data indicated that SIN1 siRNA transfection enhanced the NC-induced cell growth inhibitory effect and promoted cells more susceptible to NC.

## Discussion

Emerging evidence has demonstrated that SIN1 plays an oncogenic role in human cancer.^12^, ^13^, ^14^ For example, tumor suppressor programmed cell death 4 (Pdcd4) attenuates SIN1 translation, leading to the inhibition of cell invasion in colon carcinoma.^12^ One study revealed that the activation of SIN1 promotes cell proliferation and metastasis through the regulation of the epithelial-mesenchymal transition (EMT) in NSCLC (non-small-cell lung carcinoma) cells.^13^ In line with this report, SIN1 was found to promote invasion and metastasis via facilitating the EMT in hepatocellular carcinoma.^15^ Another study showed that SIN1 promoted proliferation and migration via the activation of Akt in breast cancer cells.^14^ Consistently, SIN1 is upregulated and associated with Akt activation in medullary and aggressive papillary thyroid carcinomas.^16^ These reports indicate that the inhibition of SIN1 could be a novel therapeutic approach for cancer treatment.

Accumulating evidence has revealed that NC facilitated anti-cancer activity in a variety of human cancers.^10^ For instance, NC was reported to suppress cell growth via inhibition of the Janus kinase 1 (JAK1)-STAT3 (signal transducer and activator of transcription 3)-signaling pathway in hepatocellular carcinoma.^17^ In support of the role of NC in hepatocellular cancer, one group validated that NC inhibited tumor growth, through blocking STAT3, ERK (extracellular signal-regulated kinase), and SHH (Sonic Hedgehog) pathways, and suppressed the expression of Bcl-2 (B cell lymphoma 2), CDK4 (cyclin-dependent kinase 4), VEGF-A (vascular endothelial growth factor A), VEGFR2 (VEGF receptor 2), and Cyclin D1 in liver cancer.^18^ Moreover, the NC-mediated tumor-suppressive function in hepatocellular carcinoma cells was reported due to the upregulation of Bax, p53, and p21 and the downregulation of Bcl-2.^19^ Similarly, NC inhibited cell growth and angiogenesis through suppression of the STAT3 pathway in gastric cancer.^20^

In breast cancer, NC inhibited cell growth and induced cell-cycle arrest via the upregulation of p53, p21, Bax, cleaved caspase-9, cleaved caspase-3, and cleaved PARP (poly ADP-ribose polymerase) and the downregulation of Bcl-2.^21^ Moreover, NC retarded cell migration and invasion via targeting the Src-associated signaling pathway in breast cancer cells.^10^ Notably, NC suppressed the EMT and cancer stem cell-like features via regulation of the hedgehog pathway in breast cancer.^22^ In nasopharyngeal carcinoma cells, NC exerted its anti-tumor activity via the upregulation of p53 protein.^23^ NC also exhibited cell growth suppression, apoptosis induction, and metastasis retardation in renal cancer cells by inhibition of the Akt and ERK pathways.^24^, ^25^ In addition, similar tumor-suppressive effects of NC on colorectal cancer cells were reported due to inhibition of the ERK pathway.^26^ In ovarian cancer cells, NC treatment led to the inhibition of migration and invasion through regulation of MMP-2/9 (Matrix metalloproteinase 2/9) and Skp2 (S-phase kinase-associated protein 2).^27^, ^28^ Altogether, without a doubt, NC exerts its anti-tumor activity in various types of human cancers.

Recently, NC attenuated glioma cell growth and colony formation via the inhibition of pDok2.^29^ Liu et al.^30^ reported that NC suppressed the cell growth, migration, and invasion via targeting the PI3K-Akt-mTOR-signaling pathway in human glioblastoma cells. Specifically, NC inhibited the phosphorylation of Akt and mTOR in glioblastoma cells.^30^ It has been reported that NC inhibited cell proliferation and induced apoptosis via the upregulation of cleaved caspase-3, cleaved caspase-9, and Bax and the downregulation of Bcl-2 in osteosarcoma cells.^11^ In line with this finding, our study demonstrated that NC treatment led to cell growth inhibition and apoptosis in MG63 and U2OS cells. One group has shown that NC inhibited cell migration and invasion via regulation of the Akt-GSK-3β (Glycogen synthase kinase-3β)-Snail-signaling pathway in U2OS cells.^31^ Consistently, in the current study, our results indicate that NC treatment resulted in the inhibition of cell migration and invasion in MG63 and U2OS cells. As SIN1 is an important oncoprotein in human cancers, the inhibition of SIN1 could be a new therapeutic approach for treating cancer. Our study identified that NC could be an effective inhibitor of SIN1 in osteosarcoma. However, deeper investigation is required to explore whether NC may exert anti-cancer activity in a mouse model and clinical trial via the inhibition of SIN1.

## Materials and Methods

### Cell Culture and Reagents

Human osteosarcoma cell lines (MG63 cells and U2OS cells) were purchased from the Chinese Academy of Science Cell Bank (Shanghai, China). Cells were cultured in DMEM containing 10% fetal bovine serum and 1% streptomycin and penicillin at 37°C in a 5% CO_2_ humidified atmosphere. NC was purchased from Tauto Biotech (Shanghai, China). NC was dissolved in DMSO to make a 20-mM stock solution, and it was added directly to the medium at different concentrations. Anti-Sin1 (12860, 1:1,000) was bought from Cell Signaling Technology (Danvers, MA, USA). Lipofectamine 2000 was bought from Invitrogen (Carlsbad, CA, USA).

### Cell Viability Assay

MG63 cells and U2OS cells were cultured in 96-well plates (5 × 10^3^cells/well) for overnight incubation, and then they were treated with different concentrations of NC for 72 h. Cell proliferation was assessed by MTT assay. Each value was normalized to cells treated with DMSO as a control group.

### Cell Apoptosis Assay

MG63 cells and U2OS cells (3 × 10^5^cells/well) were seeded in a 6-well plate overnight and then treated with various concentrations of NC for 48 h. Cells were treated with trypsinizion and washed with PBS, then resuspended in 500 μL binding buffer containing 1 μL FITC-conjugated anti-Annexin V antibody and 5 μL PI. Fluorescence-activated cell sorting (FACS) was performed to analyze the apoptotic rate.

### Cell Wound Healing Assay

The wound healing assay was performed as previously described.^32^ Briefly, MG63 cells and U2OS cells were cultured in 6-well plates and incubated at 37°C. After cells were almost confluent, the straight scratch wound was established with a 100 μL sterile pipette tip. After we removed the supernatant cells and washed with PBS, cells were treated with different concentrations of NC for 20 h. The scratched areas were photographed using a microscope at 0 and 20 h, respectively.

### Transwell Migration and Invasion Assay

The invasion capacity of MG63 and U2OS cells was determined using 24-well plate Transwell chambers with 8-μm pore size and Matrigel, as reported previously.^33^ The migration was measured in Transwell chambers without coating Matrigel. The cells treated with NC or Sin1 transfection or the combination were suspended in 200 μL serum-free DMEM and then placed on the upper chamber. 800 μL complete medium (10% fetal bovine serum [FBS]) was added in the lower chamber. After 24 h, the cells that had migrated to the underside of the membrane were stained with Crystal Violet. At least five random images were taken under a microscope, and we counted the average number of stained cells that represented the relative invasion.

### Transfection

MG63 cells and U2OS cells were grown in 6-well plates, and transfected with SIN1 cDNA, SIN1 siRNA, or empty vector using Lipofectamine 2000, according to the manufacturer’s instructions. The SIN1 siRNA was purchased from GenePharma (Shanghai, China). The SIN1 cDNA construct was obtained from Addgene (Cambridge, MA, USA).

### Western Blotting Analysis

MG63 cells and U2OS cells were harvested after NC treatment and total proteins from cells were extracted. Protein concentrations were measured by BCA (bicinchoninic acid) method. Proteins (40 μg) were separated using 10% SDS-PAGE and transferred onto polyvinylidene flouride (PVDF) membranes. The membranes were blocked with 5% non-fat milk in TBST (Tris-buffered saline with Tween) at room temperature for 2 h, and then they were incubated with primary antibodies overnight at 4°C, rinsed with TBST 3 times, and incubated with secondary antibody for 1 h at room temperature. The proteins were visualized with enhanced chemiluminescence.

### Statistical Analysis

All data were analyzed using GraphPad Prism 6.0 software. The results were expressed as the mean ± SD. Differences between each group of values and its control were evaluated by Student’s t test. Differences in four groups were analyzed by ANOVA followed by Tukey’s post hoc test. p < 0.05 was considered statistically significant.

## Author Contributions

H.X. performed the experiments, analyzed the data, and wrote the manuscript. T.C., X.Z., and Y.S. designed and performed the experiments and analyzed the data. Q.Z., S.C., L.Y., G.J., and J.M. analyzed the data. P.W. and Y.L. conceived the work, wrote the manuscript, and critically viewed and supervised the study.

## Conflicts of Interest

There are no conflicts of interest.
